# Proposal for a London Female Sanitary Society and Savings' Bank

**Published:** 1857-10

**Authors:** 


### PROPOSAL FOR A LONDON FEMALE SANITARY SOCIETY AND SAVINGS' BANK.

Mr. Acton favours us with the outline of a scheme for extending the advantages of Friendly Societies and Benefit Clubs to the unfortunates of society. That some great remedial work is wanted in this direction there is no doubt, and we admire Mr. Acton's courage in speaking out so boldly as he has done. But we reserve for future consideration any opinion as to the merits of his suggestion.

